# Autophagy Contributes to the Rapamycin-Induced Improvement of Otitis Media

**DOI:** 10.3389/fncel.2021.753369

**Published:** 2022-01-28

**Authors:** Daoli Xie, Tong Zhao, Xiaolin Zhang, Lihong Kui, Qin Wang, Yuancheng Wu, Tihua Zheng, Peng Ma, Yan Zhang, Helen Molteni, Ruishuang Geng, Ying Yang, Bo Li, Qing Yin Zheng

**Affiliations:** ^1^Hearing and Speech Rehabilitation Institute, College of Special Education, Binzhou Medical University, Yantai, China; ^2^Department of Otolaryngology-Head and Neck Surgery, Binzhou Medical University Hospital, Binzhou, China; ^3^Department of Genetics, School of Pharmacy, Binzhou Medical University, Yantai, China; ^4^Department of Otolaryngology, Head and Neck Surgery, Second Affiliated Hospital, Xi’an Jiaotong University School of Medicine, Xi’an, China; ^5^Department of Otolaryngology, Head and Neck Surgery, Case Western Reserve University, Cleveland, OH, United States

**Keywords:** otitis media, TLR2, autophagy, PGPS, rapamycin

## Abstract

Otitis media (OM) is a pervasive disease that involves hearing loss and severe complications. In our previous study, we successfully established a mouse model of human OM using *Tlr2tm1Kir* (TLR2^–/–^) mice with middle ear (ME) inoculation of streptococcal peptidoglycan-polysaccharide (PGPS). In this study, we found that hearing loss and OM infections in OM mice were significantly alleviated after treatment with rapamycin (RPM), a widely used mechanistic target of RPM complex 1 (mTORC1) inhibitor and autophagy inducer. First of all, we tested the activity of mTORC1 by evaluating p-S6, Raptor, and mTOR protein expression. The data suggested that the protein expression level of p-S6, Raptor and mTOR are decreased in TLR2^–/–^ mice after the injection of PGPS. Furthermore, our data showed that both the autophagosome protein LC3-II, Beclin-1, ATG7, and autophagy substrate protein p62 accumulated at higher levels in mice with OM than in OM-negative mice. The expression of lysosomal-associated proteins LAMP1, Cathepsin B, and Cathepsin D increased in the OM mice compared with OM-negative mice. Rab7 and Syntaxin 17, which is necessary for the fusion of autophagosomes with lysosomes, are reduced in the OM mice. In addition, data also described that the protein expression level of p-S6, mTOR and Raptor are lower than PGPS group after RPM treatment. The accumulation of LC3-II, Beclin-1, and ATG7 are decreased, and the expression of Rab7 and Syntaxin 17 are increased significantly after RPM treatment. Our results suggest that autophagy impairment is involved in PGPS-induced OM and that RPM improves OM at least partly by relieving autophagy impairment. Modulating autophagic activity by RPM may be a possible effective treatment strategy for OM.

## Introduction

OM (otitis media), one of the most common childhood infections (Rovers et al., 2004; Monasta et al., 2012), is associated with the potential burden of hearing loss and leads to excessive antibiotic consumption and severe complications (Vergison et al., 2010). The pathogenesis of OM is associated with many factors, including immune system dysfunction, genetic susceptibility, pathogen exposure, and middle ear (ME) damage (Rovers et al., 2004). ME is part of a functional system composed of the nasopharynx and eustachian tube anteriorly and the mastoid air cells posteriorly (Bluestone and Doyle, 1988). The tympanic membrane serves as the boundary between the ME and the outer ear. OM is a common inflammatory response in diseases of the auditory system (Harmes et al., 2013). Toll receptors (TLR) play a role in the innate immune response in mammals and TLR2 recognizes components from a variety of microbial pathogens (Takeda and Akira, 2015). Previously, we successfully established a mouse model of OM using *Tlr2tm1Kir* (TLR2^–/–^) mice. TLR2^–/–^ mice were inoculated with streptococcal peptidoglycan-polysaccharide (PGPS) into their ME by tympanic membrane puncture (Zhang et al., 2015). Our results demonstrated that compared with wild-type (WT) C57BL/6J mice, TLR2^–/–^ mice inoculated with PGPS exhibited severe and long-lasting inflammation and tissue damage.

Recently, autophagy was found to be involved in immunological and inflammatory diseases. Autophagy provides a source of peptides for antigen presentation and is involved in the engulfment and degradation of intracellular pathogens, and it is also a key regulator of inflammatory cytokines (Harris et al., 2017). Autophagy plays an important role in inflammasome activation and in the release of interleukin-1 (IL-1) family cytokines, which are an essential part of innate and adaptive immune responses (Garlanda et al., 2013). Autophagy is a lysosome-dependent intracellular degradation pathway unique to eukaryotes. It is considered to have several stages: autophagy induction, autophagosome formation, the fusion of autophagosomes and lysosomes and substrate degradation (Mizushima, 2007; Glick et al., 2010). The lipidated form of microtubule-associated protein 1A/1B-light chain 3 (LC3), LC3-II, and the accumulation of the cargo receptor of autophagosomes, sequestosome 1 (SQSTM1/p62), have been used as markers for active autophagy (Tanida et al., 2004; Sou et al., 2006). In recent years, researchers have discovered that the expression of mRNA associated with autophagy, like *LC3-II* and *Beclin-1* in the ME fluid samples of patients with OM increased (Jung et al., 2020a,b), but the mechanism of autophagy involved in OM has not been clarified. Therefore, our experiment would like to verify whether autophagy is involved in OM and explore the role of autophagy in OM.

Rapamycin (RPM), an autophagy inducer, activates autophagy by repressing the mechanistic target of RPM complex 1 (mTORC1) (Sekiguchi et al., 2012). It has been used in some clinical trials, such as allograft rejection, cancer and lymphangioleiomyomatosis (Bissler et al., 2008; Li et al., 2014; Franz et al., 2016; Koenig et al., 2018; Mandrioli et al., 2018). Topical RPM appears effective and safe for treatment of tuberous sclerosis complex -related facial angiofibromas. To date, RPM has been also shown to be effective in a variety of inflammatory diseases in animal model. It relieved inflammation in experimental autoimmune encephalomyelitis in a mouse model (Li et al., 2019a,b). It also suppressed airway inflammation and inflammatory molecules in retinal inflammation (Okamoto et al., 2016; Joean et al., 2017). In addition, it was shown to protect cartilage endplates from chronic inflammation-induced degeneration (Zuo et al., 2019). However, previous studies have not determined whether RPM treatment has an otoprotective effect in OM. We speculate that autophagy may be involved in the process of OM, and RPM may have a protective effect on OM. Therefore, we also want to find out whether RPM could relieves the OM caused by PGPS and identify the possible mechanism of this process.

We have confirmed that the ME inflammation of TLR2^–/–^ mice injected with PGPS was more severe than that of WT mice through the electric otoscope image and the hematoxylin-eosin (H&E) staining firstly (Supplementary Figures 1A,B). In addition, we found that the protein expressions of LC3-II and p62 were increased obviously from the ME tissues injected by PGPS in TLR2^–/–^ mice but not in WT mice, which suggested that the autophagy impairment may be involved in PGPS-induced OM in TLR2^–/–^ mice (Supplementary Figure 1C). PGPS induces relatively stable OM in TLR2^–/–^ mice, which provides a longer time window for drug screening and studying mechanisms of prevention and treatment. Based on the above reasons, we chose TLR2^–/–^ mice as our OM model. In this study, TLR2^–/–^ mice with PGPS-induced OM are called “OM mice.”

In this study, we observed the therapeutic effect of RPM against PGPS-induced OM and investigated the role of autophagy in this process. Our results suggested that RPM alleviates hearing loss and inflammation in the OM mice and that normal autophagy contributes to this process. We hope that our study will help improve the clinical treatment of OM.

## Materials and Methods

### Animals

Both male and female *Tlr2tm1Kir* (TLR2^–/–^) mice and WT mice aged 6–8 weeks were obtained from the Jackson Laboratory (Bar Harbor, ME, United States) and housed in a pathogen-free facility. The experimental protocol was approved by the Animal Use and Care Committee of Binzhou Medical University.

### Drug Treatment

Mice were treated with normal saline (NS), PGPS, NS + DMSO, or PGPS + rapamycin (RPM). Individual mice were intraperitoneally anesthetized with 4% chloral hydrate (0.01 ml/g; Biotopped Life Sciences, Beijing, China). All the mice received treatment in their right ears. The mice in the NS group received intratympanic (IT) injections of 10 μl of saline. The mice in the PGPS group received IT injections of 60 μg PGPS (100P, BD Bioscience, San Jose, CA, United States) freshly prepared in 10 μl of NS as described previously (Zhang et al., 2015). Purified (purity 99%) PGPS was extracted from the *Streptococcus pneumoniae* cell wall (Fulghum and Brown, 1998; Komori et al., 2011). RPM (Selleck, S1039, Shanghai, China) for IT injections was dissolved in 100% dimethyl sulfoxide (DMSO) to make 22 mM stock solutions and diluted with a PGPS solution immediately before injection for a final dose of either 0.35 or 0.7 μM in a 10 μl PGPS solution. 0.7 μM RPM and 0.35 μM are obtained from 22 mM stock solutions diluted with PBS. The mice in the vehicle group received IT injections of equal volumes of DMSO or NS. The mouse tympanic membranes were examined on day 3 post-injection using an otoscopic digital imaging system (MedRx VetScope System, Largo, FL, United States).

### Auditory Brainstem Response and Tympanometry Procedure

The Auditory brainstem response (ABR) and tympanometry of individual experimental mice were assessed on day 3 post-injection. A computer-aided evoked potential system (IHS3.30 Intelligent Hearing Systems, Miami, FL, United States) was used for ABR measurements as described previously (Hu et al., 2016). Briefly, click, 8, 16, and 32 kHz tone burst frequencies were channeled through an earphone inserted into the right ear. The ABR threshold was identified as the lowest stimulus level at which clear and repeatable waveforms were recognized. Tympanometry measurements were performed using an MT 10 tympanometer (Interacoustics, Assens, Denmark).

### Histological Analysis of the Middle Ear

The experimental mice were sacrificed on day 3 post-injection, and their right auditory bullae (including both the middle and inner ear) were dissected and subjected to pathological examination as described previously (Zhang et al., 2015). The bullae tissues were fixed with 4% paraformaldehyde for 24 h at 4°C, decalcified with a 10% EDTA solution for 5 days, and embedded in paraffin. The paraffin sections were stained with H&E and examined under a light microscope (Leica DMI4000 B, Germany).

### Immunohistochemistry

The right bullae from experimental mice were fixed in 4% paraformaldehyde and decalcified before they were embedded in paraffin. The bullae tissues were sectioned at 5–7 μm. After deparaffinization, rehydration, and antigen retrieval, the sections were immunohistochemically stained with an anti-p62 antibody (Abcam, ab56416, Cambridge, England, United Kingdom), anti-Beclin-1 antibody (Abcam, ab210498), anti-ATG7 antibody (Proteintech, 10088-2-AP), anti-Cathepsin B Rabbit Polyclonal Antibody (Proteintech, 12216-1-AP), anti-Cathepsin D antibody (Proteintech, 21327-1-AP), anti-Rab7 antibody (Abcam, ab137029), anti-p-S6 antibody (Ser235/236) (Cell Signaling Technology, #4858), anti-mTOR antibody (Proteintech, 20657-1-AP), and anti-Raptor antibody (Affinity, #DF7527). The sample slides were observed under a light microscope and imaged by LAS X software (Leica DM4500 B, Leica Microsystems Inc., Buffalo Grove, IL, United States).

### Immunofluorescence

After deparaffinization, rehydration, and antigen retrieval, the sections were stained with an anti-LC3B antibody (Novus Biologicals, NB100-2220, Co., United States), anti-TNF-α antibody (Proteintech, 17590-1-AP), anti-LAMP1 antibody (Abcam, ab24170), anti-Syntaxin 17 antibody (Proteintech, 17815-1-AP) and DAPI (Invitrogen, Carlsbad, United States). The stained tissues were imaged using a confocal microscope (LSM 880, Zeiss, Oberkochen, Germany).

### Terminal Deoxynucleotidyl Transferase-Mediated dUTP-Biotin Nick End Labeling Staining

Paraffin sections from bullae tissues obtained from the mice were stained using a TUNEL Kit (*In Situ* Cell Death Detection Kit, Fluorescein, 11684795910; Roche Diagnostics) and following the manufacturer’s protocol. The samples were then viewed under a fluorescence microscope (Leica DM4500 B, Leica Microsystems Inc., Buffalo Grove, IL, United States).

### Statistical Analysis

Each experiment was repeated at least three times. All the data are presented as the mean ± SEM. Data analyses were conducted using Microsoft Excel and GraphPad Prism 9 software (GraphPad, San Diego, CA, United States). Unpaired Student’s *t*-tests were used to determine the statistical significance when comparing two groups, and one-way ANOVA was used when comparing more than two groups. The value of *P* < 0.05 was considered statistically significant.

## Results

### PGPS Induces Severe Otitis Media

**FIGURE 1:**
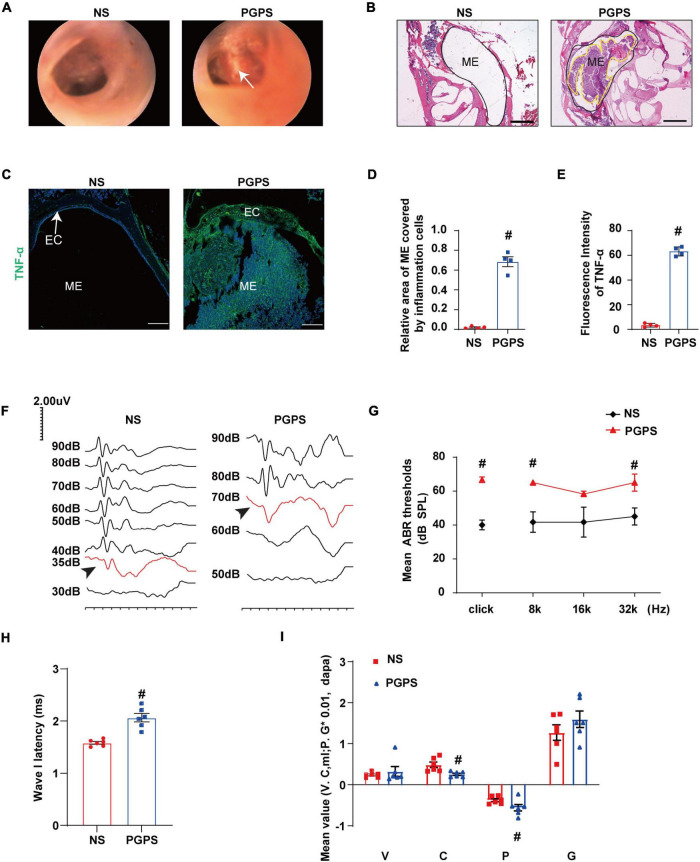
Inoculation with PGPS induces ME inflammation and hearing loss. **(A)** Otoscopic images of ears from the NS and PGPS groups are shown. Hyperemia and hydrotympanum (white arrow) were detected in PGPS group. **(B)** H&E histology showing the structures and pathology of the ME. **(C)** Mice were inoculated with NS or PGPS for 3 days. Representative immunostaining for TNF-α expression in ME. **(D)** Quantification of the relative area of ME covered by inflammatory cells is shown in the bar graph. *n* = 4 per group **(E)** Quantification of the fluorescence intensity of TNF-α is shown in the bar graph. *n* = 4 per group **(F)** Representative images of ABR waveforms at click stimuli are shown. The red lines and arrowheads represent threshold waveforms. **(G)** Mice were inoculated with PGPS for 3 days, and the ABR thresholds were measured at the stimuli frequencies of click, 8 kHz, 16 kHz, and 32 kHz. The mean ABR thresholds in PGPS-injected mice were compared with those in NS-injected mice. The data is presented as the mean ± SEM. *n* = 10 per group **(H)** The latency of ABR wave I at click stimuli (80 dB SPL) in PGPS-injected mice compared with that in NS-injected mice is shown. Horizontal bars are mean values. *n* = 10 per group. **(I)** The tympanometry values in PGPS-injected mice compared with those in NS-injected mice are shown. The data are presented as the mean ± SEM. *n* = 10 per group. V represents the mean value of volume, C represents compliance in tympanometry parameters, G represents the gradient, and P represents the pressure. ^#^*P* < 0.05 vs. NS group, Student’s *t*-test. Scales bar, 100 μm **(B,C)**.

TLR2^–/–^ mice were inoculated with 10 μl of 60 μg PGPS or NS solution. Images of the mouse ears under an otoscope showed that hyperemia and hydrotympanum (white arrowhead) were present in the ears of the PGPS group (Figure 1A). Histological examination revealed excessive inflammatory infiltrations in the tympanic cavity and severe tissue damage in the PGPS group compared with the NS group (Figure 1B). The inflammatory areas in the ME of PGPS-treated mice were significantly larger than those of NS-treated mice (Figure 1D). Tumor Necrosis Factor α (TNF-α), an inflammatory cytokine, is responsible for a diverse range of signaling events within cells, leading to necrosis or apoptosis (Idriss and Naismith, 2000). The immunofluorescence staining revealed that expression levels of TNF-α increased in the PGPS group (Figures 1C,E). Taken together, these data confirmed that PGPS induced severe inflammation in the mouse ME.

Next, we investigated the hearing function of the OM mice. The average ABR thresholds and tympanometry values were measured after inoculation. Representative images of ABR waveforms for click stimuli are shown in Figure 1F. The mean ABR thresholds in the PGPS group were significantly higher than those in the NS solution group at click, 8 kHz and 32 kHz stimulus frequencies (Figure 1G). The latency of ABR wave I at click stimuli (80 dB SPL) increased in PGPS-injected mice compared with NS-injected mice (Figure 1H). There were significant differences in tympanometry value, compliance, and pressure between the NS and PGPS groups (Figure 1I). These data indicated that the OM mice developed severe hearing impairment.

### Rapamycin Treatments Alleviate the Severity of Otitis Media Induced by PGPS

**FIGURE 2:**
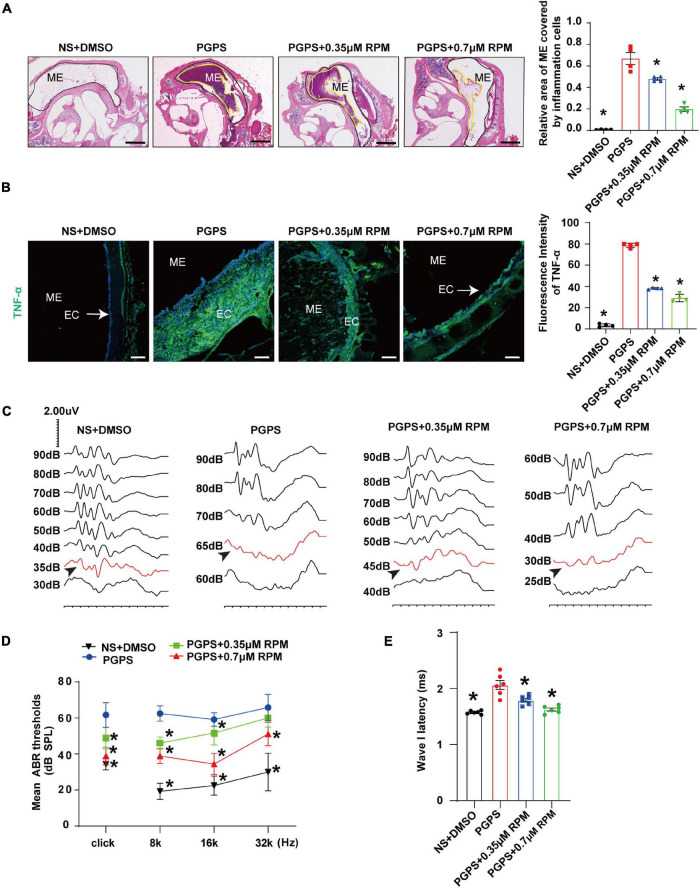
Rapamycin treatment inhibits PGPS-induced inflammation and improves hearing function in PGPS-inoculated OM mice. **(A)** H&E histological images showing the structures and pathology of the ME. Quantification of the relative area of ME covered by inflammatory cells is shown in the bar graph. *n* = 4 per group. **(B)** Representative immunostaining for TNF-α expression in the ME. Quantification of the fluorescence intensity of TNF-α is shown in the bar graph. *n* = 4 per group. **(C)** Representative images of ABR waveforms at click stimuli are shown. The red lines and arrowheads represent threshold waveforms. **(D)** Mice were inoculated with PGPS or a combination of PGPS and either 0.35 or 0.7 μM RPM. The ABR thresholds were measured at the stimuli frequencies of click, 8, 16, and 32 kHz. The mean ABR thresholds in the NS + DMSO, PGPS + 0.35 μM RPM, and PGPS + 0.7 μM RPM groups were compared with those in the PGPS group. The data is presented as the mean ± SEM. *n* = 10 per group. **(E)** The latency of ABR wave I at click stimuli (80 dB SPL) in the NS + DMSO, PGPS + RPM combination treatment groups were compared with that in the PGPS group. Horizontal bars are mean values. *n* = 10 per group. *P < 0.05 vs. the PGPS group, one-way ANOVA, Scale bar, 100 μm **(A)**, 50 μm **(B)**.

Previous studies have shown that RPM improves inflammation in organs such as the airway and retina. Thus, we investigated whether RPM has a protective effect in PGPS-induced OM mice. Histomorphological examination showed that after RPM treatment, the ME inflammation was reduced, and the inflammation area of ME in RPM-treated mice was significantly smaller than that of PGPS-treated mice (Figure 2A). In addition, the immunofluorescence staining revealed that the expression of TNF-α decreased more in the RPM-treated mice than in the PGPS-treated mice (Figure 2B). These results showed that RPM could reduce the inflammatory infiltrates in the tympanic cavity and the expression level of TNF-α. Representative images of ABR waveforms at click stimuli are shown in Figure 2C. The mean ABR thresholds decreased more in the PGPS + 0.35 μM RPM group and the PGPS + 0.7 μM RPM group than in the PGPS-treated group at click, 8 and 16 kHz stimulus frequencies (Figure 2D). The latency of ABR wave I at click stimuli (80 dB SPL) decreased in the PGPS + 0.7 μM RPM group compared to the PGPS-treated group (Figure 2E). These data suggested that RPM may ease hearing loss by attenuating PGPS-induced inflammation in OM mice.

Taking into account the complexity of RPM signaling pathways, we also injected the mice with 0.35 and 0.7 μM RPM separately. Compared with the PGPS group, the two groups of mice injected with RPM alone had normal morphology and no obvious inflammatory cells (Supplementary Figure 2A). The results of immunofluorescence demonstrated that the expression of TNF-α was almost invisible after the RPM injection alone (Supplementary Figure 2B). Compared with the PGPS group, the area of ME covered by inflammatory cells and TNF-α expression level were significantly lower in the RPM injection alone group. Compared with the NS group, there was no statistical significance. The experimental results showed that there was no obvious inflammation caused by RPM injection alone.

We also investigated the hearing function in RPM single treatment group by ABR, and the representative images of ABR waveforms at click stimuli are shown in Supplementary Figure 2C. Compared with the PGPS group, the average ABR thresholds of the NS group, 0.35 and 0.7 μM RPM group at click, 8, 16, and 32 kHz stimulation frequencies were lower than those in the PGPS group, and were statistically significant (Supplementary Figure 2D). In addition, the latency of ABR wave I in the PGPS group under the click stimulus (80 dB SPL) was longer than that of the other three groups (Supplementary Figure 2E). Compared with NS group, the hearing threshold and the wave I latency in RPM treatment alone group is basically no statistical significance. These data showed that after injection of RPM alone, there is almost no effect on the hearing of mice.

### Autophagy Impairment Is Involved in PGPS-Induced Otitis Media

**FIGURE 3:**
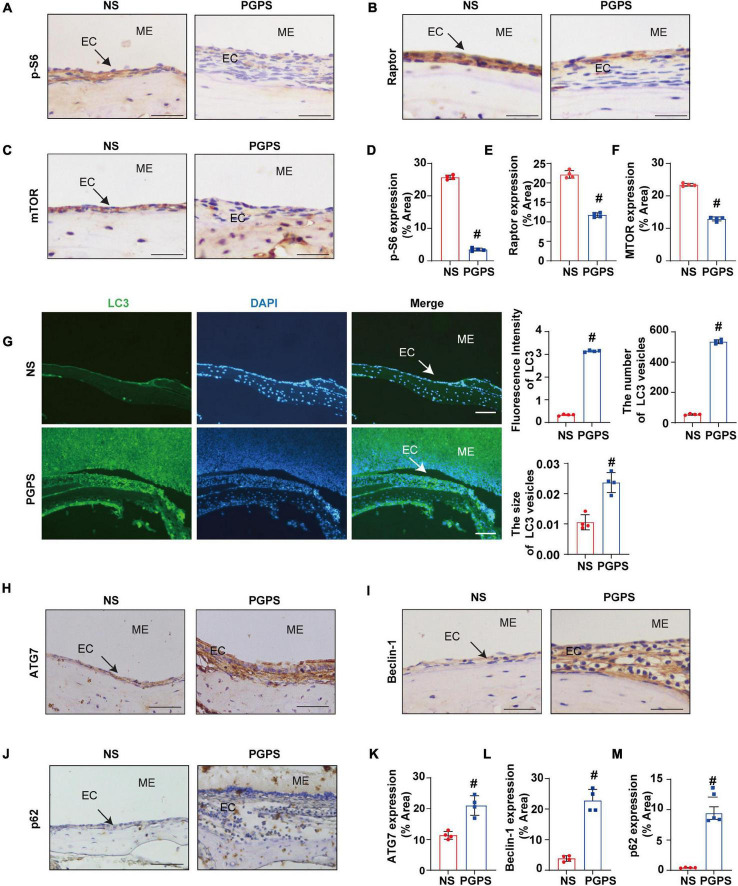
Peptidoglycan-polysaccharide induces autophagy impairment in OM mice. Mice were inoculated with NS or PGPS for 3 days. Paraffin-embedded sections of ME tissues were immunostained with antibodies. **(A)** The representative images of p-S6 expression in ME tissues. **(B)** The representative images of Raptor expression in ME tissues. **(C)** The representative images of mTOR expression in ME tissues. **(D)** The quantification of p-S6 expression in ME tissues. **(E)** The quantification of Raptor expression in ME tissues. **(F)** The quantification of mTOR expression in ME tissues. **(G)** The representative images of LC3 expression, as well as quantitative images of the fluorescence intensity of LC3 protein expression and quantitative images of the size and number of LC3 vesicles. **(H)** The representative images of ATG7 expression in ME tissues. **(I)** The representative images of Beclin-1 expression in ME tissues. **(J)** The representative images and quantification of p62 expression in ME tissues. **(K)** The quantification of ATG7 expression in ME tissues. **(L)** The quantification of Beclin-1 expression in ME tissues. **(M)** The quantification of p-62 expression in ME tissues. ^#^*P* < 0.05 vs. NS group, *n* = 4 per group, Student’s *t*-test. ME represents the middle ear; EC represents epithelial cells. Scale bar = 25 μm.

Rapamycin, an autophagy inducer, activates autophagy by repressing the mTORC1 (Sekiguchi et al., 2012). S6 ribosomal protein (S6) phosphorylation was shown to be a critical downstream component of mTOR signaling (Ruvinsky et al., 2005). mTORC1 contains mTOR, which is the catalytic subunit of the complex (Laplante and Sabatini, 2009). It also contains the large protein Raptor, which is the regulatory-associated protein of mTOR (Thoreen et al., 2009). To find out whether mTORC1 signaling is involved in PGPS-induced autophagy impairment, we examined the phosphorylation of mTORC1 substrate, S6 phosphorylation at 235/236 (p-S6) and mTORC1 components, mTOR and Raptor. PGPS treatment resulted in a significant decrease in the levels of p-S6, Raptor and mTOR compared with NS group (Figures 3A–F), suggesting an inhibition of mTORC1 activity. In this study, we found that the fluorescence intensity of LC3 in PGPS-treated OM mice was higher than that in NS-treated mice. Quantification of the size and number of LC3 vesicles also increased in PGPS-treated OM mice (Figure 3G). These results suggested that OM mice are activated at the initial stage of autophagy, but it may also be due to the accumulation of LC3 caused by the blocked autophagy flux.

In addition, we also tested the expressions of ATG7 and Beclin-1. ATG7 is considered to be essential molecules for the induction of autophagy (Arakawa et al., 2017), and Beclin-1 initiates the nucleation step of autophagy to begin autophagic flux (Liang et al., 1999; Matsunaga et al., 2009). The results of immunohistochemistry showed that compared with the NS group, the expression of ATG7 and Beclin-1 were increased in OM mice (Figures 3H,I,K,L). These results suggested that after the injection of PGPS, the activity of mTORC1 was inhibited and the initial stage of autophagy was activated. We speculate that autophagy may act as an instinctive stress response to resist external stimuli by PGPS. However, p62 protein accumulation in the ME epithelial cells of OM mice was higher after PGPS treatment than after NS treatment (Figures 3J,M). These data indicated that PGPS could induce the initiation of autophagy, but at the same time cause impairment in the degradation of stage autophagy.

### PGPS Induces Dysfunction of Autophagosome and Lysosome Fusion

**FIGURE 4:**
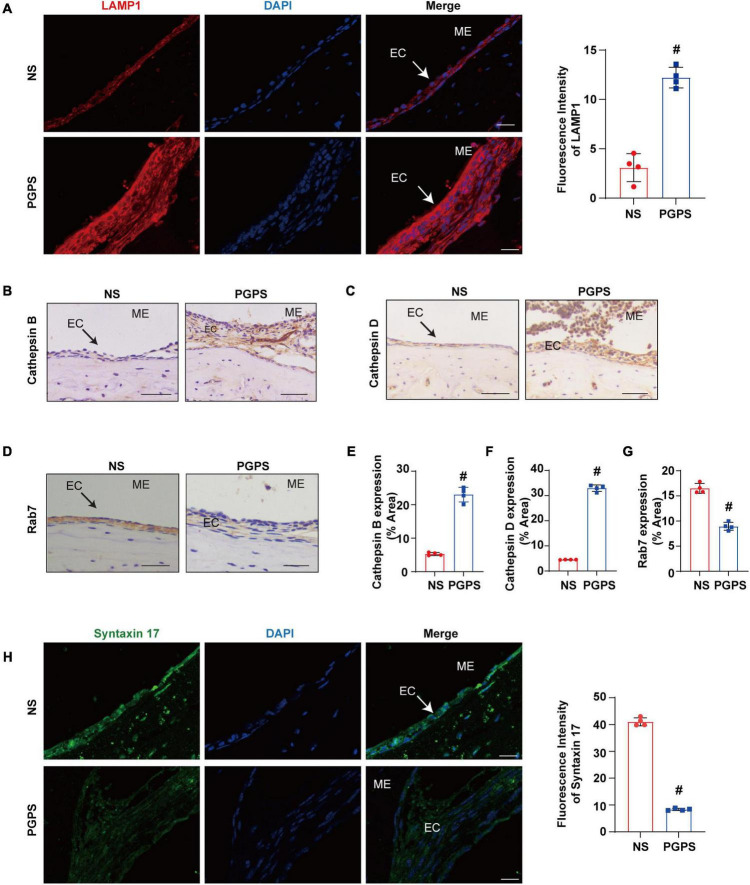
Peptidoglycan-polysaccharide induces obstacles to the fusion of autophagosomes and lysosomes in OM mice. Mice were inoculated with NS or PGPS for 3 days. Paraffin-embedded sections of ME tissues were immunostained with antibodies. **(A)** The representative images and quantification of LAMP1 expression in ME tissues. **(B)** The representative images of Cathepsin B expression in ME tissues. **(C)** The representative images of Cathepsin D expression in ME tissues. **(D)** The representative images of Rab7 expression in ME tissues. **(E)** The quantification of Cathepsin B expression in ME tissues. **(F)** The quantification of Cathepsin D expression in ME tissues. **(G)** The quantification of Rab7 expression in ME tissues. **(H)** The representative images and quantification of Syntaxin 17 expression in ME tissues. ^#^*P* < 0.05 vs. NS group, *n* = 4 per group, Student’s *t*-test. ME represents the middle ear; EC represents epithelial cells. Scale bar = 25 μm.

The dysfunction of autophagy degradation may be due to the impairment of lysosomal function or dysfunction in the fusion stage of autophagosomes and lysosomes. Firstly, we test lysosomal function. Lysosomal activity is important for the autophagy degradation process (Tai et al., 2017). In order to investigate whether the autophagy impairment mechanism induced by PGPS is related to the dysfunction of lysosome, we examined the protein expression level of key lysosome enzymes like LAMP-1, Cathepsin B and Cathepsin D to evaluate the lysosomal function. Lysosome associated membrane protein-1 (LAMP-1) is a major protein component of the lysosomal membrane (Eskelinen, 2006). Cathepsin B, a member of the cysteine cathepsin family, involved in regulating the bioavailability of lysosomes and autophagosomes (Man and Kanneganti, 2016). Cathepsin D is one of the major lysosomal proteases indispensable for the maintenance of cellular proteostasis (Marques et al., 2020). Immunofluorescence results showed that compared with NS group, the expression of LAMP1 protein increased in the PGPS group (Figure 4A). The immunohistochemical results of Cathepsin B and Cathepsin D also showed a consistent increase in PGPS group (Figures 4B,C,E,F). These data showed that lysosome function maybe is not impaired.

Considering that lysosome function does not seem to be impaired, we examined the process of autophagosome and lysosome fusion by evaluating expression of Rab7 and Syntaxin 17 protein. Rab7 is a member of the Rab family, involved in transport to late endosomes and in the biogenesis of the perinuclear lysosome compartment (Gutierrez et al., 2004; Guerra and Bucci, 2016). It plays a critical role in the final maturation of late autophagic vacuoles (autophagosome and lysosome fusion) (Jager et al., 2004; He et al., 2019). Syntaxin 17 is also required for fusion between the autophagosome and lysosome (Itakura et al., 2012; Shen et al., 2021). Our results showed that Rab7 and Syntaxin 17 expression decreased in the PGPS group compared with the NS group (Figures 4D,G,H). These results suggested that PGPS may block the autophagy degradation stage mainly due to the impairment of the autophagosome and lysosome fusion stage. And PGPS may impair the fusion of autophagosomes with lysosomes by decreasing the expression of Rab7 and Syntaxin 17.

### Rapamycin Treatment Enhances Autophagy in PGPS-Treated Otitis Media Mice

**FIGURE 5:**
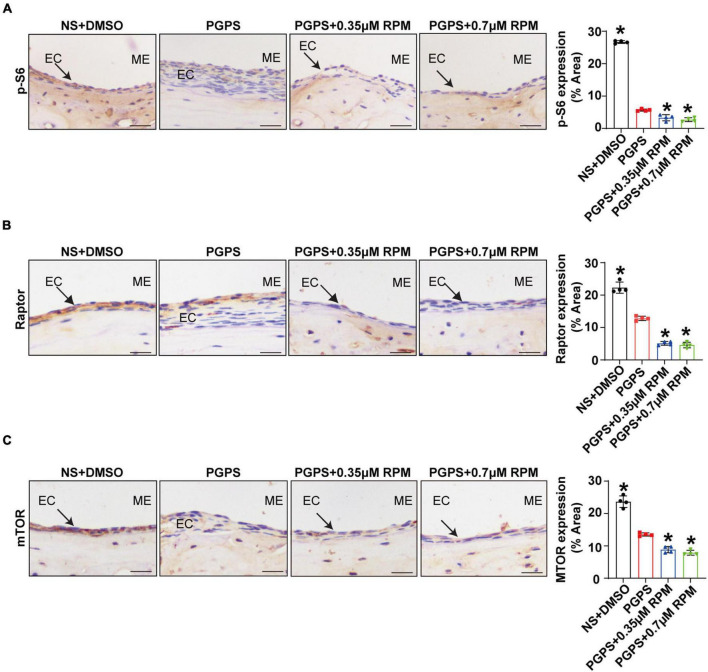
Rapamycin treatment inhibits mTORC1 activity. Mice were inoculated with PGPS or a combination of PGPS and either 0.35 or 0.7 μM RPM. Paraffin-embedded sections of ME tissues were immunostained antibodies. **(A)** The representative images and quantification of p-S6 expression in ME tissues. **(B)** The representative images and quantification of Raptor expression in ME tissues. **(A)** The representative images and quantification of p-S6 expression in ME tissues. **(C)** The representative images and quantification of mTOR expression in ME tissues. *n* = 4 per group. ME represents the middle ear; EC represents epithelial cells. *P < 0.05 vs. the PGPS group, one-way ANOVA, Scale bar = 25 μm.

**FIGURE 6:**
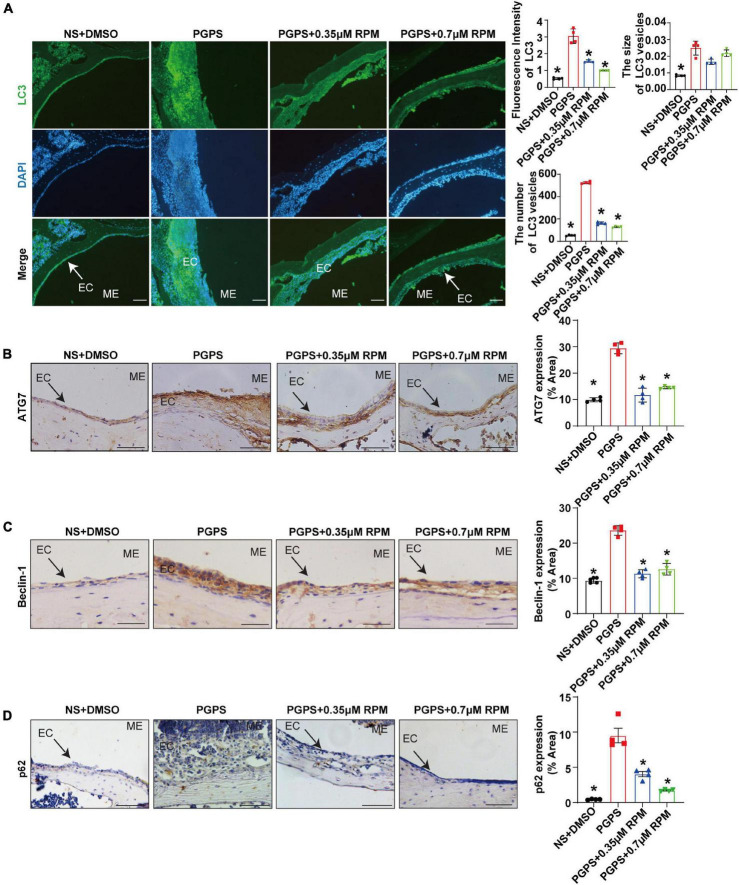
Rapamycin treatment enhances autophagy in PGPS-treated OM mice. Mice were inoculated with PGPS or a combination of PGPS and either 0.35 or 0.7 μM RPM. Paraffin-embedded sections of ME tissues were immunostained antibodies. **(A)** The representative images of LC3 expression, as well as quantitative images of the fluorescence intensity of LC3 protein expression and quantitative images of the size and number of LC3 vesicles. *n* = 4 per group. **(B)** The representative images and quantification of ATG7 expression in ME tissues. *n* = 4 per group. **(C)** The representative images and quantification of Beclin-1 expression in ME tissues. *n* = 4 per group. **(D)** The representative images and quantification of p62 expression in ME tissues. *n* = 4 per group. ME represents the middle ear; EC represents epithelial cells. *P < 0.05 vs. the PGPS group, one-way ANOVA, Scale bar = 25 μm.

Immunohistochemical staining showed that after injection of PGPS + 0.35/0.7 μM RPM, p-S6, Raptor and mTOR exhibited lower protein levels than the PGPS group (Figures 5A–C). These results suggested that RPM may enhance autophagic initiation by inhibiting mTORC1 activity. LC3 staining in ME epithelial cells revealed lower expression of LC3 and lower numbers of LC3 vesicles in RPM-treated mice than in PGPS-treated mice, the size of LC3 vesicles did not show a significant difference (Figure 6A). These results suggested that there may be a certain accumulation of LC3 after injection of PGPS, and after the treatment of RPM, the autophagy flux may became smooth. Immunohistochemical staining showed that after injection of PGPS + 0.35/0.7 μM RPM, ATG7 and Beclin-1 exhibited lower protein levels than the PGPS group (Figures 6B–C).

In order to understand the role of RPM, we injected RPM alone in TLR2^–/–^ mice. The results showed that the expression of LC3 and the number of LC3 vesicles in RPM injection group was lower than that of the PGPS group, but it was higher than that of the NS group (Supplementary Figures 3A,B,D). There was almost no statistical difference in the size of LC3 vesicles among the groups (Supplementary Figures 3A,C). These results suggested that after the injection of PGPS, the mouse ME epithelial cells activated the initiation of autophagy to resist the toxicity by PGPS. However, obstacle may occur in the autophagy degradation stage, which led to the accumulation of LC3 protein. Moreover, RPM may promote the degradation stage of autophagy.

In addition, there was less p62 protein accumulation in the RPM-treated mice than in the PGPS-treated mice (Figure 6D). After injection of RPM alone, the expression level of p62 was significantly lower compared with the PGPS group, and there was almost no difference compared with the NS group (Supplementary Figure 3E). These results indicated that degradation function may be improved after RPM treatment.

**FIGURE 7:**
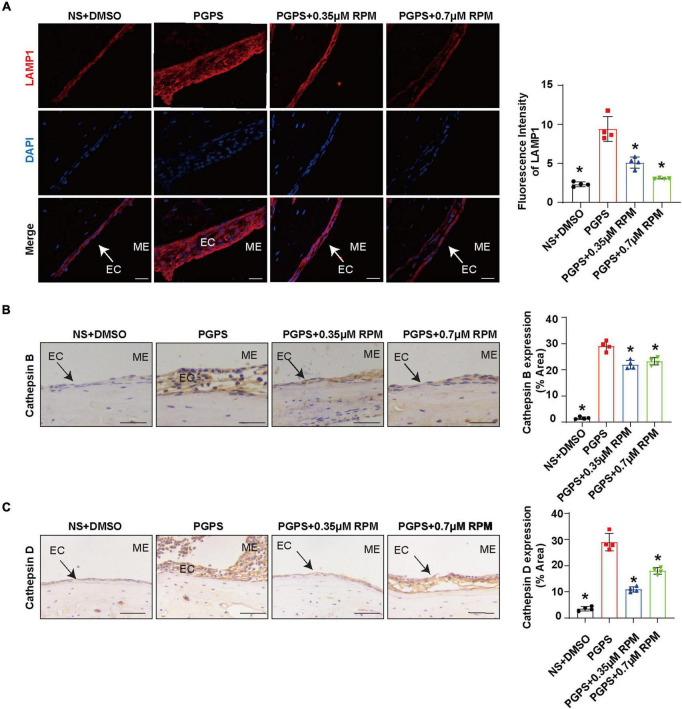
Lysosome function may not be impaired after PGPS injection Mice were inoculated with PGPS or a combination of PGPS and either 0.35 or 0.7 μM RPM. Paraffin-embedded sections of ME tissues were immunostained antibodies. **(A)** The representative images and quantification of LAMP1 expression in ME tissues. **(B)** The representative images and quantification of Cathepsin B expression in ME tissues. **(C)** The representative images and quantification of Cathepsin D expression in ME tissues. *n* = 4 per group, ME represents the middle ear; EC represents epithelial cells. *P < 0.05 vs. the PGPS group, one-way ANOVA, Scale bar = 25 μm.

**FIGURE 8:**
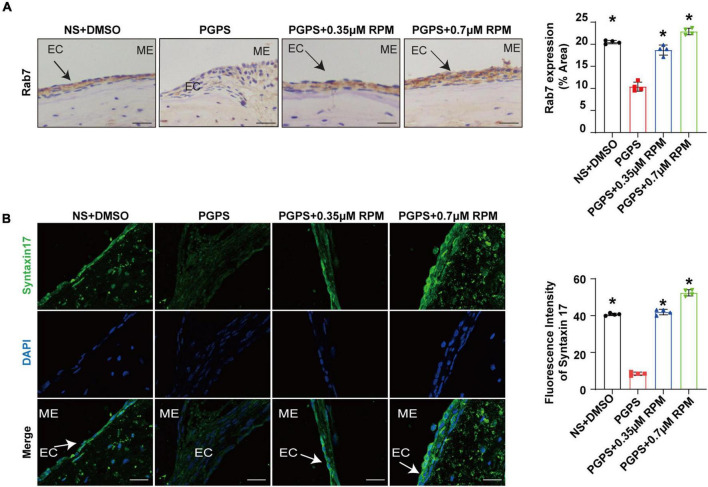
Rapamycin treatment increases the fusion of autophagosomes and lysosomes in OM mice. Mice were inoculated with PGPS or a combination of PGPS and either 0.35 or 0.7 μM RPM. Paraffin-embedded sections of ME tissues were immunostained antibodies. **(A)** The representative images and quantification of Rab7 expression in ME tissues. **(B)** The representative images and quantification of Syntaxin 17 expression in ME tissues. *n* = 4 per group, ME represents the middle ear; EC represents epithelial cells. *P < 0.05 vs. the PGPS group, one-way ANOVA, Scale bar = 25 μm.

**FIGURE 9:**
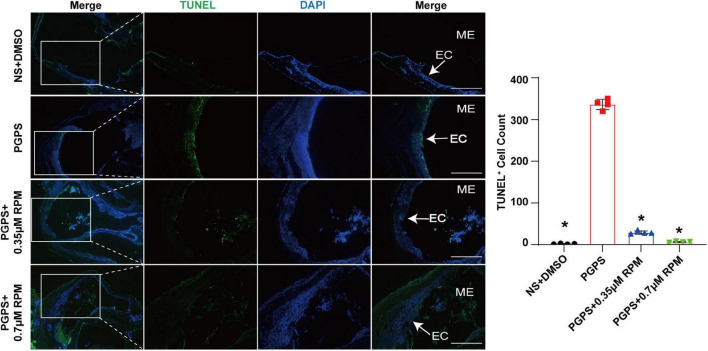
Rapamycin treatment relieved ME epithelial cell apoptosis. Apoptotic cells in the MEs were examined by TUNEL staining. The quantitative image of apoptotic cells was shown in Figure B. The PGPS + 0.7 μM RPM group showed fewer TUNEL-positive epithelial cells than the PGPS group. ME represents the middle ear; EC represents epithelial cells. *P < 0.05 vs. the PGPS group, *n* = 4 per group, Scale bar = 500 μm, one-way ANOVA.

Similarly, we tested lysosome function and the fusion function of autophagosome and lysosome after RPM treatment alone. The results showed that the expression of LAMP1, Cathepsin B and Cathepsin D (Figures 7A–C) in the RPM-treated mice was relatively weaker than that in the PGPS group. After RPM injection alone, we found that the expression level of Cathepsin B protein was lower than that of the PGPS group, but higher than that of the NS group (Supplementary Figure 3F). We further speculate that the increased activity of lysosomal after PGPS injection may be a response to external stimuli by PGPS, so the expression level of Cathepsin B is higher than that of the RPM injection group. We speculate that RPM may promote lysosome function, thus the expression level of Cathepsin B in RPM injection alone group is higher than that of NS group. In addition, the expression of Rab7 and Syntaxin 17 protein increased in the PGPS + RPM-treated mice (Figures 8A,B), thus RPM may promote the process of autophagosome and lysosome fusion. TUNEL staining showed that there were less apoptotic cells in the ME after RPM treatment (Figure 9). In addition, we did not find obvious apoptotic cells in the RPM alone group (Supplementary Figure 4). These data indicated that RPM may enhance the autophagic activity of OM mice by inhibiting the activity of mTORC1, increasing the fusion of autophagosomes with lysosomes and relieving ME epithelial cell apoptosis.

## Discussion

Otitis media, a general term for inflammatory changes in the ME cavity, is one of the most common childhood conditions (Mittal et al., 2014; Venekamp et al., 2017). The pathogenic mechanism of OM is not yet clear and excessive antibiotic treatment has also brought a heavy burden to society, so it is particularly important to explore the mechanism of OM and find suitable drug treatments (Vergison et al., 2010). In recent years, the autophagy pathway has played a certain role in inflammation, but the research on the relationship between autophagy and OM has not been in-depth. It was found that the expression of *LC3-II* was significantly increased in the inflammatory ME tissues in human (Jung et al., 2020b). Studies have shown that in the ME fluid of patients with OM, the mRNA level of autophagy initiation-related genes such as *Beclin-1*, is increased in OM patients with cholesteatom (Jung et al., 2020a). Our study aims to explore the role of autophagy in the pathogenesis of OM by TLR2^–/–^ mice model, and treat OM mice by RPM, aiming to provide a theoretical basis and new treatment strategies for the treatment of clinical OM.

The mTOR is involved in the induction and initiation of autophagy (Cayo et al., 2021). We tested the expression of p-S6, Raptor and mTOR after injection of PGPS, and found that the mTORC1 activity of mice in the PGPS group was weakened. These results indicated that autophagy may be activated in the mice in the PGPS group, which seems to be consistent with the increase in the expression of proteins related to autophagy initiation like LC3, ATG7 and Beclin-1. Among them, the expressions of LC3, ATG7 and Beclin-1 in the PGPS group all showed increased compared with NS group. We speculate that autophagy may act as an instinctive stress response to resist external stimuli by PGPS. RPM + PGPS combination treatment groups showed that the protein expression of LC3, ATG7 and Beclin-1 were reduced compared with the PGPS group. Considering the complexity of the RPM pathway, we also injected RPM alone in TLR2^–/–^ mice. The results suggested that the expression of LC3, ATG7, and Beclin-1 in RPM injection alone group was also lower than PGPS group, but was higher than NS group. These results indicated that the PGPS group does not seem to be impaired during the initiation of autophagy. We speculated that there may be obstacles in the degradation stage of autophagy, resulting in accumulation of LC3 protein in PGPS group. The expression of p62 in PGPS group is more increasing than NS group, and after the injection of PGPS + RPM combination, the level of p62 decreased, which demonstrated that PGPS may induce autophagy impairment in the autophagy degradation stage and RPM may promote the degradation stage of autophagy.

Both the function of lysosome and the fusion of autophagosomes and lysosomes affect the autophagic degradation. We first evaluated the lysosomal function. Lysosome-related proteins such as LAMP1, Cathepsin B and Cathepsin D play an important role in the normal function of lysosomes (Eskelinen, 2006; Man and Kanneganti, 2016; Marques et al., 2020). In our experiment, compared with the NS group, the expression of the three proteins increased in the PGPS group. The expression level of the three proteins in the RPM + PGPS combination treatment group was lower than that of the PGPS group. After injection of RPM alone, the expression of Cathepsin B also decreased compared with PGPS group, but was higher than NS group. These results seemed to demonstrate that the lysosomes of OM mice are not impaired. On the contrary, increased lysosomal function may be a stress response after PGPS injection.

Based on the above experimental results, we tested the fusion stage of autophagosomes and lysosomes by evaluating the protein expression of Rab7 and Syntaxin 17. Our results showed that after PGPS injection, the expression of Rab7 and Syntaxin 17 decreased, and co-treatment with RPM, the expression of Rab7 and Syntaxin 17 increased. These results suggested that after injection of PGPS, the fusion of autophagosome and lysosome is impaired, leading to autophagy impairment. RPM treatment may stimulate the fusion of autophagosomes and lysosomes, making the autophagy pathway smoothly. Previous study also found that RPM could promote the fusion of lysosomes and autophagosomes (Choi et al., 2016). In addition, we also found that after PGPS and RPM combination treatment, the expression of p-S6, mTOR and Raptor was lower than that of PGPS alone. Therefore, we speculate that when TLR2^–/–^ mice injected with PGPS, autophagy acts as an instinctive stress response to resist external stimuli. But, there may be an obstacle when autophagosomes fuse with lysosomes, which leads to obstacles in the degradation stage of autophagy and causes protein accumulation. This may produce proteotoxic stress, and autophagy is insufficient to resist proteotoxic stress during OM. After RPM treatment, mTORC1 could be further inhibited, thereby promoting the initial stage of autophagy. At the same time, our experimental results also found that RPM may promote the degradation stage of autophagy. In summary, RPM may play a positive role in both stages, so as to exert its therapeutic effect.

**FIGURE 10:**
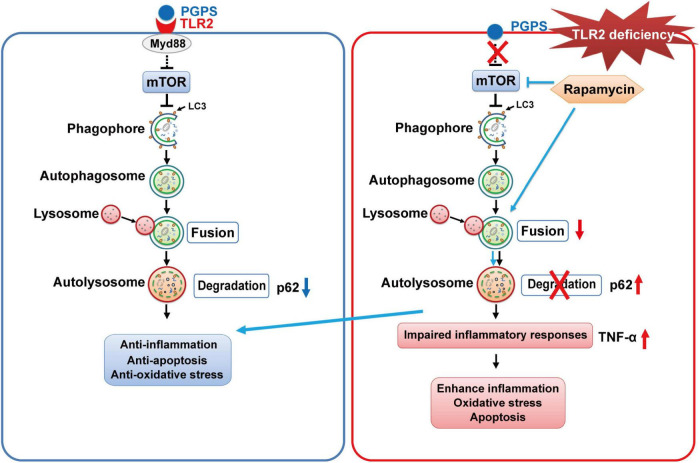
The proposed mechanism for RPM-enhanced autophagy protects against PGPS-induced OM in TLR2^– /–^ mice. TLR2 deficiency may inhibit fusion between autophagosomes and lysosomes, leading to the accumulation of p62. It may also induce inflammation and apoptosis. RPM treatment may improve the autophagic clearance ability and have a protective effect against OM injury.

In this study, we verify again that PGPS injection could cause OM in TLR2^–/–^ mice, which leads to hearing loss. In addition, we demonstrated that inflammation in OM mice may be due to the impairment of autophagy pathway, mainly due to impairment in the process of autophagosome fusing to lysosomes, which is manifested by the decrease of Rab7 and Syntaxin 17 expression in the PGPS group. We also found that after injection of RPM, it could inhibit the activity of mTORC1, increase the expression level of Rab7 and Syntaxin 17 and promote autophagy flux. RPM treatment also reduce the inflammatory response of ME epithelial cells, reduce cell apoptosis, and thereby alleviate hearing loss in OM mice. Therefore, these data suggested that autophagy impairment may be involved in the development of OM and that RPM could effectively improve OM conditions, most likely by alleviating autophagy impairment. A general scheme showing that RPM-enhanced autophagy protects against PGPS-induced OM in TLR2^–/–^ mice is shown in Figure 10.

Autophagy is a bulk degradation system that delivers cytoplasmic constituents to autolysosomes for recycling and maintaining cell homeostasis. In addition, autophagy has critical functions in cell-autonomous defense in immunity (Matsuzawa-Ishimoto et al., 2018). Many studies found autophagy impairment could mediate susceptibility to infectious and inflammatory diseases like Crohn’s disease and chronic obstructive pulmonary disease (Lupfer et al., 2013; Lassen et al., 2014; Murthy et al., 2014). In this study, we found for the first time that autophagy was involved in OM and that RPM significantly alleviated autophagy impairment and improved ME inflammatory conditions.

To date, RPM has been used in some clinical trials like tuberous sclerosis complex-related facial angiofibromas (Koenig et al., 2018). It has been also shown to be effective in a variety of inflammatory diseases like autoimmune encephalomyelitis and retinal inflammation (Okamoto et al., 2016; Joean et al., 2017; Li et al., 2019a,b). In this study, we showed that RPM significantly alleviated autophagy impairment and improved ME inflammatory conditions. Some researchers also found that RPM could promote the fusion of lysosomes and autophagosomes (Choi et al., 2016; Cheng et al., 2021). Therefore, we speculate that RPM may play a positive role in the treatment of OM. Due to the complexity of RPM signaling pathways, we do not rule out the possibility that the protective effect of RPM on the OM may also involve other branches of RPM signaling in addition to autophagy. Nonetheless, our data suggest that modulating autophagy activity may be possible intervention for OM. Our study provides a theoretical basis for the clinical application of RPM in the treatment of OM. However, it has been reported that RPM has immunosuppressive side effects. Several new RPM analogs have demonstrated reduced side effects, and these new drugs may be safer and less immunosuppressive than RPM (Fu et al., 2018). Perhaps the ability of these analogs to prevent OM should be tested.

In summary, our research shows that autophagy impairment is related to OM, and impairment to the fusion of autophagosomes and lysosomes is an important factor leading to the occurrence of PGPS-induced otitis media. RPM treatment could alleviate hearing loss to a certain extent. These findings highlight the potential of specific autophagosome-to-lysosome fusion activators in reducing PGPS-induced OM. Considering that increasing autophagic clearance may be useful as a new therapeutic strategy against severe OM damage, autophagosome and lysosome fusing dysfunction may be a candidate target for therapeutic intervention. Therefore, extensive pharmaceutical studies should be performed in the near future. Future research is necessary to better explain the mechanism underlying the protective role of the normal autophagy process against the pathogenesis of OM, and then design and test potential therapeutic methods to prevent or treat OM.

## Data Availability Statement

The original contributions presented in the study are included in the article/Supplementary Material, further inquiries can be directed to the corresponding authors.

## Ethics Statement

The animal study was reviewed and approved by the Animal Use and Care Committee of Binzhou Medical University.

## Author Contributions

DX, ToZ, XZ, LK, QW, and YW performed the experiments. BL and DX wrote the manuscript. ToZ and BL analyzed the data. RG, YY, PM, XZ, and YZ participated in discussion of the project. BL and QZ designed the study. YW, TiZ, HM, and QZ revised the manuscript. All authors reviewed and approved the manuscript.

## Conflict of Interest

The authors declare that the research was conducted in the absence of any commercial or financial relationships that could be construed as a potential conflict of interest.

## Publisher’s Note

All claims expressed in this article are solely those of the authors and do not necessarily represent those of their affiliated organizations, or those of the publisher, the editors and the reviewers. Any product that may be evaluated in this article, or claim that may be made by its manufacturer, is not guaranteed or endorsed by the publisher.

## References

[B1] Arakawa S. Honda S. Yamaguchi H. Shimizu S. (2017). Molecular mechanisms and physiological roles of Atg5/Atg7-independent alternative autophagy. *Proc. Jpn. Acad. Ser. B Phys. Biol. Sci.* 93 378–385. 10.2183/pjab.93.023 28603209 PMC5709538

[B2] Bissler J. J. McCormack F. X. Young L. R. Elwing J. M. Chuck G. Leonard J. M. (2008). Sirolimus for angiomyolipoma in tuberous sclerosis complex or lymphangioleiomyomatosis. *N. Engl. J. Med.* 358 140–151. 18184959 10.1056/NEJMoa063564 PMC3398441

[B3] Bluestone C. D. Doyle W. J. (1988). Anatomy and physiology of eustachian tube and middle ear related to otitis media. *J. Allergy Clin. Immunol.* 81 997–1003. 10.1016/0091-6749(88)90168-6 3286738

[B4] Cayo A. Segovia R. Venturini W. Moore-Carrasco R. Valenzuela C. Brown N. (2021). mTOR activity and autophagy in senescent cells, a complex partnership. *Int. J. Mol. Sci.* 22:8149. 10.3390/ijms22158149 34360912 PMC8347619

[B5] Cheng J. T. Liu P. F. Yang H. C. Huang S. J. Griffith M. Morgan P. (2021). Tumor Susceptibility gene 101 facilitates rapamycin-induced autophagic flux in neuron cells. *Biomed. Pharmacother.* 134:111106. 10.1016/j.biopha.2020.111106 33338748

[B6] Choi J. Y. Won N. H. Park J. D. Jang S. Eom C. Y. Choi Y. (2016). From the cover: ethylmercury-induced oxidative and endoplasmic reticulum stress-mediated autophagic cell death: involvement of autophagosome-lysosome fusion arrest. *Toxicol. Sci.* 154 27–42. 10.1093/toxsci/kfw155 27511942

[B7] Eskelinen E. L. (2006). Roles of LAMP-1 and LAMP-2 in lysosome biogenesis and autophagy. *Mol. Aspects Med.* 27 495–502. 10.1016/j.mam.2006.08.005 16973206

[B8] Franz D. N. Belousova E. Sparagana S. Bebin E. M. Frost M. D. Kuperman R. (2016). Long-term use of everolimus in patients with tuberous sclerosis complex: final results from the EXIST-1 study. *PLoS One* 11:e0158476. 10.1371/journal.pone.0158476 27351628 PMC4924870

[B9] Fu X. Sun X. Zhang L. Jin Y. Chai R. Yang L. (2018). Tuberous sclerosis complex-mediated mTORC1 overactivation promotes age-related hearing loss. *J. Clin. Invest.* 128 4938–4955. 10.1172/JCI98058 30247156 PMC6205401

[B10] Fulghum R. S. Brown R. R. (1998). Purified streptococcal cell wall (PG-APS) causes experimental otitis media. *Auris Nasus Larynx* 25 5–11. 10.1016/s0385-8146(97)10025-6 9512788

[B11] Garlanda C. Dinarello C. A. Mantovani A. (2013). The interleukin-1 family: back to the future. *Immunity* 39 1003–1018. 10.1016/j.immuni.2013.11.010 24332029 PMC3933951

[B12] Glick D. Barth S. Macleod K. F. (2010). Autophagy: cellular and molecular mechanisms. *J. Pathol.* 221 3–12. 10.1002/path.2697 20225336 PMC2990190

[B13] Guerra F. Bucci C. (2016). Multiple Roles of the Small GTPase Rab7. *Cells* 5:34. 10.3390/cells5030034 27548222 PMC5040976

[B14] Gutierrez M. G. Munafo D. B. Beron W. Colombo M. I. (2004). Rab7 is required for the normal progression of the autophagic pathway in mammalian cells. *J. Cell Sci.* 117 2687–2697. 10.1242/jcs.01114 15138286

[B15] Harmes K. M. Blackwood R. A. Burrows H. L. Cooke J. M. Harrison R. V. Passamani P. P. (2013). Otitis media: diagnosis and treatment. *Am. Fam. Phys.* 88 435–440. 24134083

[B16] Harris J. Lang T. Thomas J. P. W. Sukkar M. B. Nabar N. R. Kehrl J. H. (2017). Autophagy and inflammasomes. *Mol. Immunol.* 86 10–15. 28249679 10.1016/j.molimm.2017.02.013

[B17] He K. Sun H. Zhang J. Zheng R. Gu J. Luo M. (2019). Rab7mediated autophagy regulates phenotypic transformation and behavior of smooth muscle cells via the Ras/Raf/MEK/ERK signaling pathway in human aortic dissection. *Mol. Med. Rep.* 19 3105–3113. 10.3892/mmr.2019.9955 30816458 PMC6423587

[B18] Hu J. Li B. Apisa L. Yu H. Entenman S. Xu M. (2016). ER stress inhibitor attenuates hearing loss and hair cell death in Cdh23(erl/erl) mutant mice. *Cell Death Dis.* 7:e2485. 10.1038/cddis.2016.386 27882946 PMC5260868

[B19] Idriss H. T. Naismith J. H. (2000). TNF alpha and the TNF receptor superfamily: structure-function relationship(s). *Microsc. Res. Tech.* 50 184–195. 10.1002/1097-0029(20000801)50:3&lt;184::AID-JEMT2&gt;3.0.CO;2-H 10891884

[B20] Itakura E. Kishi-Itakura C. Mizushima N. (2012). The hairpin-type tail-anchored SNARE syntaxin 17 targets to autophagosomes for fusion with endosomes/lysosomes. *Cell* 151 1256–1269. 10.1016/j.cell.2012.11.001 23217709

[B21] Jager S. Bucci C. Tanida I. Ueno T. Kominami E. Saftig P. (2004). Role for Rab7 in maturation of late autophagic vacuoles. *J. Cell Sci.* 117 4837–4848. 10.1242/jcs.01370 15340014

[B22] Joean O. Hueber A. Feller F. Jirmo A. C. Lochner M. Dittrich A. M. (2017). Suppression of Th17-polarized airway inflammation by rapamycin. *Sci. Rep.* 7:15336. 10.1038/s41598-017-15750-6 29127369 PMC5681547

[B23] Jung J. Jung S. Y. Kim M. G. Kim Y. I. Kim S. H. Yeo S. G. (2020a). Comparison of autophagy mRNA expression between chronic otitis media with and without cholesteatoma. *J. Audiol. Otol.* 24 191–197. 10.7874/jao.2020.00108 32521994 PMC7575920

[B24] Jung J. Park D. C. Kim Y. I. Lee E. H. Park M. J. Kim S. H. (2020b). Decreased expression of autophagy markers in culture-positive patients with chronic otitis media. *J. Int. Med. Res.* 48:300060520936174. 10.1177/0300060520936174 32589484 PMC7323285

[B25] Koenig M. K. Bell C. S. Hebert A. A. Roberson J. Samuels J. A. Slopis J. M. (2018). Efficacy and safety of topical rapamycin in patients with facial angiofibromas secondary to tuberous sclerosis complex: the treatment randomized clinical trial. *JAMA Dermatol.* 154 773–780. 10.1001/jamadermatol.2018.0464 29800048 PMC6128508

[B26] Komori M. Nakamura Y. Ping J. Feng L. Toyama K. Kim Y. (2011). Pneumococcal peptidoglycan-polysaccharides regulate Toll-like receptor 2 in the mouse middle ear epithelial cells. *Pediatr. Res.* 69 101–105. 10.1203/PDR.0b013e3182055237 21076367 PMC3020247

[B27] Laplante M. Sabatini D. M. (2009). mTOR signaling at a glance. *J. Cell Sci.* 122 3589–3594. 10.1242/jcs.051011 19812304 PMC2758797

[B28] Lassen K. G. Kuballa P. Conway K. L. Patel K. K. Becker C. E. Peloquin J. M. (2014). Atg16L1 T300A variant decreases selective autophagy resulting in altered cytokine signaling and decreased antibacterial defense. *Proc. Natl. Acad. Sci. U.S.A.* 111 7741–7746. 10.1073/pnas.1407001111 24821797 PMC4040621

[B29] Li J. Kim S. G. Blenis J. (2014). Rapamycin: one drug, many effects. *Cell Metab.* 19 373–379. 10.1016/j.cmet.2014.01.001 24508508 PMC3972801

[B30] Li Z. Nie L. Chen L. Sun Y. Guo L. (2019a). [Rapamycin alleviates inflammation by up-regulating TGF-beta/Smad signaling in a mouse model of autoimmune encephalomyelitis]. *Nan Fang Yi Ke Da Xue Xue Bao* 39 35–42. 10.12122/j.issn.1673-4254.2019.01.06 30692064 PMC6765580

[B31] Li Z. Nie L. Chen L. Sun Y. Li G. (2019b). Rapamycin relieves inflammation of experimental autoimmune encephalomyelitis by altering the balance of Treg/Th17 in a mouse model. *Neurosci. Lett.* 705 39–45. 10.1016/j.neulet.2019.04.035 31004709

[B32] Liang X. H. Jackson S. Seaman M. Brown K. Kempkes B. Hibshoosh H. (1999). Induction of autophagy and inhibition of tumorigenesis by beclin 1. *Nature* 402 672–676. 10.1038/45257 10604474

[B33] Lupfer C. Thomas P. G. Anand P. K. Vogel P. Milasta S. Martinez J. (2013). Receptor interacting protein kinase 2-mediated mitophagy regulates inflammasome activation during virus infection. *Nat. Immunol.* 14 480–488. 10.1038/ni.2563 23525089 PMC3631456

[B34] Man S. M. Kanneganti T. D. (2016). Regulation of lysosomal dynamics and autophagy by CTSB/cathepsin B. *Autophagy* 12 2504–2505. 10.1080/15548627.2016.1239679 27786577 PMC5173259

[B35] Mandrioli J. D’Amico R. Zucchi E. Gessani A. Fini N. Fasano A. (2018). Rapamycin treatment for amyotrophic lateral sclerosis: protocol for a phase II randomized, double-blind, placebo-controlled, multicenter, clinical trial (RAP-ALS trial). *Medicine (Baltimore)* 97:e11119. 10.1097/MD.0000000000011119 29901635 PMC6024184

[B36] Marques A. R. A. Di Spiezio A. Thiessen N. Schmidt L. Grotzinger J. Lullmann-Rauch R. (2020). Enzyme replacement therapy with recombinant pro-CTSD (cathepsin D) corrects defective proteolysis and autophagy in neuronal ceroid lipofuscinosis. *Autophagy* 16 811–825. 10.1080/15548627.2019.1637200 31282275 PMC7158922

[B37] Matsunaga K. Saitoh T. Tabata K. Omori H. Satoh T. Kurotori N. (2009). Two Beclin 1-binding proteins, Atg14L and Rubicon, reciprocally regulate autophagy at different stages. *Nat. Cell Biol.* 11 385–396. 10.1038/ncb1846 19270696

[B38] Matsuzawa-Ishimoto Y. Hwang S. Cadwell K. (2018). Autophagy and Inflammation. *Annu. Rev. Immunol.* 36 73–101. 29144836 10.1146/annurev-immunol-042617-053253

[B39] Mittal R. Kodiyan J. Gerring R. Mathee K. Li J. D. Grati M. (2014). Role of innate immunity in the pathogenesis of otitis media. *Int. J. Infect. Dis.* 29 259–267. 10.1016/j.ijid.2014.10.015 25447732 PMC4310697

[B40] Mizushima N. (2007). Autophagy: process and function. *Genes Dev.* 21 2861–2873. 10.1101/gad.1599207 18006683

[B41] Monasta L. Ronfani L. Marchetti F. Montico M. Vecchi Brumatti L. Bavcar A. (2012). Burden of disease caused by otitis media: systematic review and global estimates. *PLoS One* 7:e36226. 10.1371/journal.pone.0036226 22558393 PMC3340347

[B42] Murthy A. Li Y. Peng I. Reichelt M. Katakam A. K. Noubade R. (2014). A Crohn’s disease variant in Atg16l1 enhances its degradation by caspase 3. *Nature* 506 456–462. 10.1038/nature13044 24553140

[B43] Okamoto T. Ozawa Y. Kamoshita M. Osada H. Toda E. Kurihara T. (2016). The neuroprotective effect of rapamycin as a modulator of the mTOR-NF-kappaB axis during retinal inflammation. *PLoS One* 11:e0146517. 10.1371/journal.pone.0146517 26771918 PMC4714903

[B44] Rovers M. M. Schilder A. G. Zielhuis G. A. Rosenfeld R. M. (2004). Otitis media. *Lancet* 363 465–473. 14962529 10.1016/S0140-6736(04)15495-0

[B45] Ruvinsky I. Sharon N. Lerer T. Cohen H. Stolovich-Rain M. Nir T. (2005). Ribosomal protein S6 phosphorylation is a determinant of cell size and glucose homeostasis. *Genes Dev.* 19 2199–2211. 10.1101/gad.351605 16166381 PMC1221890

[B46] Sekiguchi A. Kanno H. Ozawa H. Yamaya S. Itoi E. (2012). Rapamycin promotes autophagy and reduces neural tissue damage and locomotor impairment after spinal cord injury in mice. *J. Neurotrauma* 29 946–956. 21806471 10.1089/neu.2011.1919

[B47] Shen Q. Shi Y. Liu J. Su H. Huang J. Zhang Y. (2021). Acetylation of STX17 (syntaxin 17) controls autophagosome maturation. *Autophagy* 17 1157–1169. 10.1080/15548627.2020.1752471 32264736 PMC8143222

[B48] Sou Y. S. Tanida I. Komatsu M. Ueno T. Kominami E. (2006). Phosphatidylserine in addition to phosphatidylethanolamine is an in vitro target of the mammalian Atg8 modifiers, LC3, GABARAP, and GATE-16. *J. Biol. Chem.* 281 3017–3024. 10.1074/jbc.M505888200 16303767

[B49] Tai H. Wang Z. Gong H. Han X. Zhou J. Wang X. (2017). Autophagy impairment with lysosomal and mitochondrial dysfunction is an important characteristic of oxidative stress-induced senescence. *Autophagy* 13 99–113. 10.1080/15548627.2016.1247143 27791464 PMC5240829

[B50] Takeda K. Akira S. (2015). Toll-like receptors. *Curr. Protoc. Immunol.* 109 14.12.1–14.12.10. 10.1002/0471142735.im1412s109 25845562

[B51] Tanida I. Ueno T. Kominami E. (2004). LC3 conjugation system in mammalian autophagy. *Int. J. Biochem. Cell Biol.* 36 2503–2518. 10.1016/j.biocel.2004.05.009 15325588 PMC7129593

[B52] Thoreen C. C. Kang S. A. Chang J. W. Liu Q. Zhang J. Gao Y. (2009). An ATP-competitive mammalian target of rapamycin inhibitor reveals rapamycin-resistant functions of mTORC1. *J. Biol. Chem.* 284 8023–8032. 10.1074/jbc.m900301200 19150980 PMC2658096

[B53] Venekamp R. P. Damoiseaux R. A. Schilder A. G. (2017). Acute Otitis Media in Children. *Am Fam. Phys.* 95 109–110. 28084706

[B54] Vergison A. Dagan R. Arguedas A. Bonhoeffer J. Cohen R. Dhooge I. (2010). Otitis media and its consequences: beyond the earache. *Lancet Infect. Dis.* 10 195–203. 10.1016/S1473-3099(10)70012-8 20185098

[B55] Zhang X. Zheng T. Sang L. Apisa L. Zhao H. Fu F. (2015). Otitis media induced by peptidoglycan-polysaccharide (PGPS) in TLR2-deficient (Tlr2(-/-)) mice for developing drug therapy. *Infect. Genet. Evol.* 35 194–203. 10.1016/j.meegid.2015.08.019 26296608 PMC4617670

[B56] Zuo R. Wang Y. Li J. Wu J. Wang W. Li B. (2019). Rapamycin induced autophagy inhibits inflammation-mediated endplate degeneration by enhancing Nrf2/Keap1 signaling of cartilage endplate stem cells. *Stem Cells* 37 828–840. 10.1002/stem.2999 30840341

