# Supplementary material for: Autophagy Contributes to the Rapamycin-Induced Improvement of Otitis Media
Source: Front Cell Neurosci. 2022 Jan 28;15:753369. doi: 10.3389/fncel.2021.753369 (PMC8832103; doi:10.3389/fncel.2021.753369)
Supplement: Supplementary file 1 [file Data_Sheet_1.PDF]

Supplementary Material

1 Supplementary Methods

Western Blot

The bullae tissues were lysed with RIPA lysis and an extraction buffer (ThermoFisher Scientific, 89900). Protein extracts (30–50 μg) were separated on polyacrylamide gels and transferred to polyvinylidene fluoride membranes (Merck Millipore, IPVH00010) for 40 minutes at 15 V using a semi-dry transfer system Bio-Rad, Hercules, CA, USA). The membranes were blocked with 5% nonfat-dried milk and then probed overnight at 4℃ with relevant primary antibodies: anti-LC3B (Novus Biologicals, NB100-2220), anti-p62 (Abcam, ab56416) and anti-GAPDH (Proteintech, 10494-1-AP). After washing with Tris-Buffered Saline-Tween 20 (TBS-T), membranes were probed again for 1 hour at room temperature with a species-specific secondary antibody coupled with horseradish peroxidase. The western blot bands were detected using a Chemiluminescent HRP Substrate kit (Merck Millipore, WBKLS0100) and visualized using a ChemiDoc XRS+ System (Bio-Rad, Hercules, CA, USA). The intensities of protein bands were measured and quantified using ImageJ software from the National Institutes for Health, as described by Miller (http://lukemiller.org/journal/2007/08/quantifying-western-blots-without.html).

2 Supplementary Figures Legend

Supplementary Figure 1.

TLR2$^{-/-}$ mice were selected as our OM model. (A) Images of the mouse ears under an otoscope showed that hyperemia and hydrotympanum (white arrowhead) were present in the ears of the PGPS group. (B) Histomorphology images showed the pathology of the ME in WT mice. (C) The protein expressions of LC3 and p62 were increased from the ME tissues in TLR2$^{-/-}$mice. The right panel shows the results of densitometric analysis. $^{\#}P < 0.05$ vs NS group, n = 4 per group, student's *t*-tests.

Supplementary Figure 2.

Injection of RPM alone did not affect the hearing of mice. (A) H&E histological images showed the structures and pathology of the ME. The inflammatory areas in the ME of RPM-treated mice alone (0.35μM RPM or 0.7μM RPM) were significantly smaller than those of mice treated with PGPS. Quantification of the relative area of ME covered by inflammatory cells is shown in the bar graph. n = 4 per group **(B)** Representative immunostaining for TNF-α expression in the ME. Quantification of the fluorescence intensity of TNF-α is shown in the bar graph. n= 4 per group **(C)** Representative images of ABR waveforms at click stimuli are shown. The red lines and arrowheads represent threshold waveforms. n = 10 per group **(D)** The ABR thresholds were measured at the stimuli frequencies of click, 8 kHz, 16 kHz, and 32 kHz. The mean ABR thresholds in the NS+DMSO, 0.35 μM RPM and 0.7 μM RPM groups were compared with those in the PGPS group. The data is presented as the mean ± SEM. n = 10 per group **(E)** The latency of ABR wave I at click stimuli (80 dB SPL) in the NS+DMSO, 0.35 μM RPM, and 0.7 μM RPM groups were compared with that in the PGPS group. n = 6 per group. Horizontal bars are mean values. $^{*}P < 0.05$ vs the PGPS group, $^{\#}P < 0.05$ vs NS group, one-way ANOVA. Scale bar, 100 μm (A). 50 μm (B).

Supplementary Figure 3.

Injection of RPM promoted autophagy flux. Mice were inoculated with NS+DMSO, PGPS, 0.35 μM or 0.7 μM RPM alone. Paraffin-embedded sections of ME tissues were immunostained antibodies. **(A)** The representative images of LC3 expression in ME tissues. **(B)** Quantification of the fluorescence intensity of LC3 is shown in the bar graph. **(C)** Quantification of the size of LC3 vesicles. **(D)** Quantification of the number of LC3 vesicles. **(E)** The representative images and quantification of p62 expression in ME tissues. **(F)** The representative images and quantification of Cathepsin B expression in ME tissues. $^{*}P < 0.05$ vs the PGPS group, $^{\#}P < 0.05$ vs NS group, n = 4 per group, one-way ANOVA. ME represents the middle ear; EC represents epithelial cells. Scale bar = 25 μm.

Supplementary Figure 4.

RPM injection alone does not cause apoptosis. Apoptotic cells in the MEs were examined by TUNEL staining (A). The quantitative image of apoptotic cells was shown in Figure B. The 0.35 μM RPM and 0.7 μM RPM group showed fewer TUNEL-positive epithelial cells than the PGPS group. ME represents the middle ear; EC represents epithelial cells. $^{*}P < 0.05$ vs the PGPS group, $^{\#}P < 0.05$ vs NS group, n = 4 per group, Scale bar = 50 μm, one-way ANOVA.
