# Supplementary figures and images for: Autophagy Contributes to the Rapamycin-Induced Improvement of Otitis Media
Source: Front Cell Neurosci. 2022 Jan 28;15:753369. doi: 10.3389/fncel.2021.753369 (PMC8832103; doi:10.3389/fncel.2021.753369)

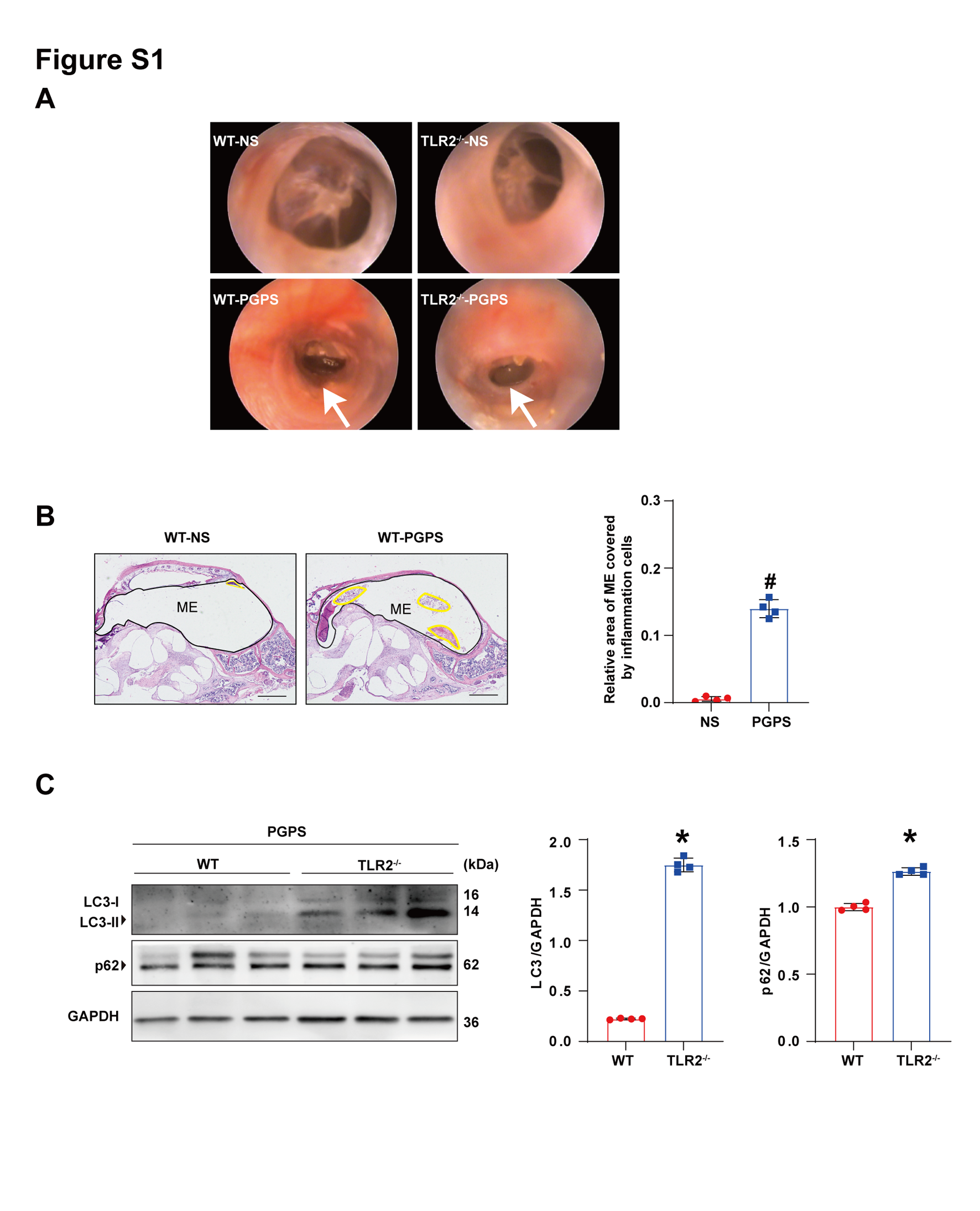

Supplement: Supplementary file 2 [file Image_1.TIF]

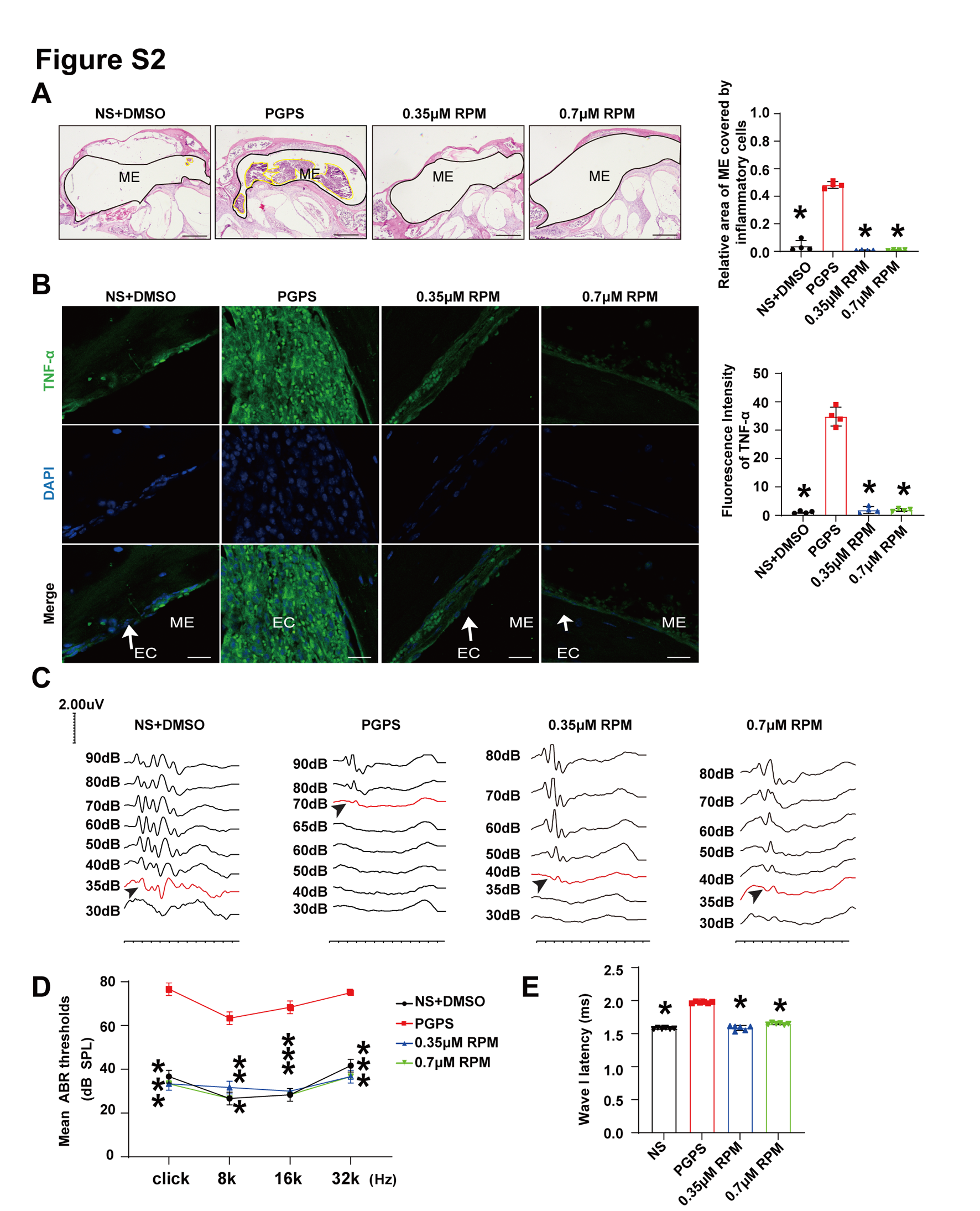

Supplement: Supplementary file 3 [file Image_2.TIF]

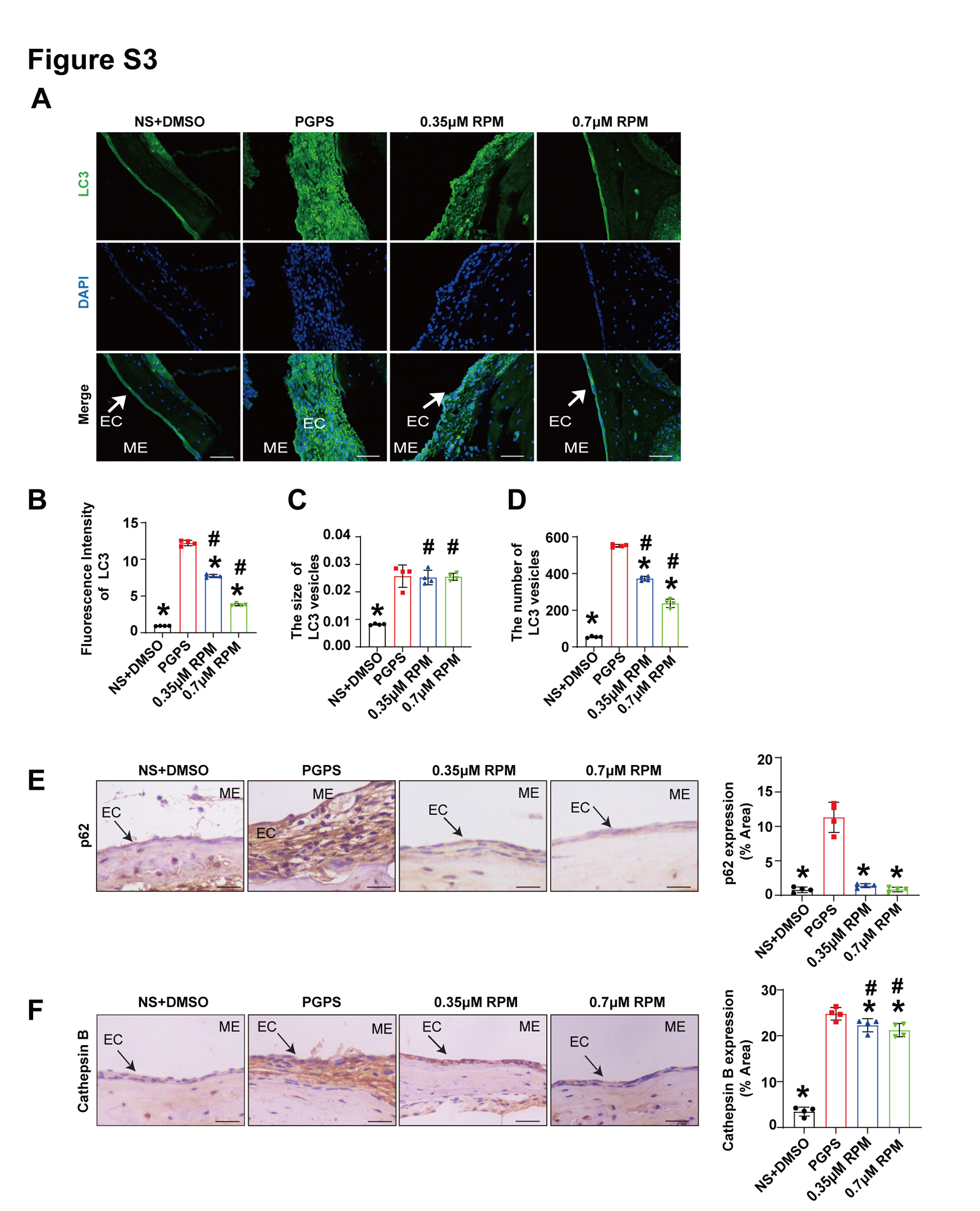

Supplement: Supplementary file 4 [file Image_3.TIF]

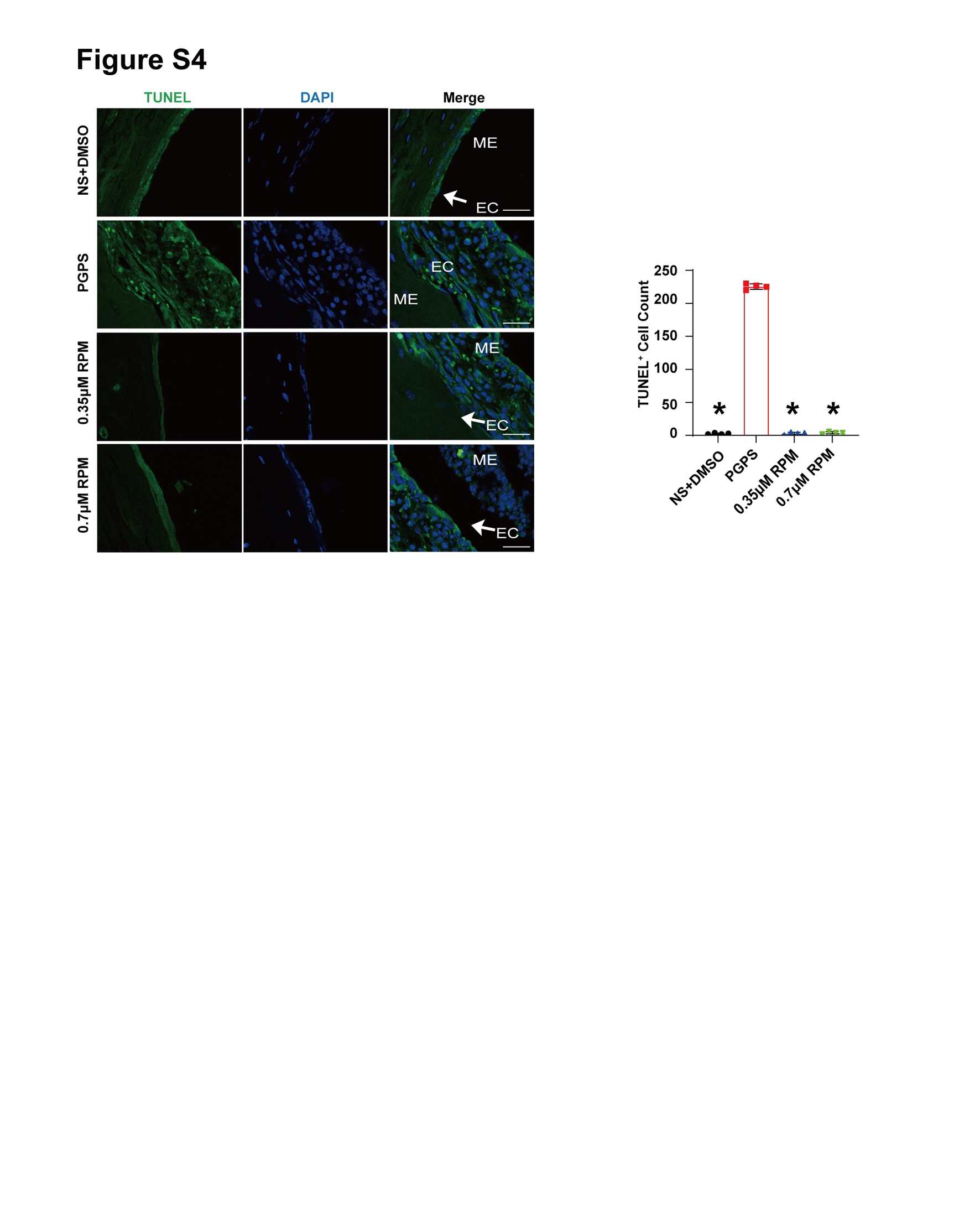

Supplement: Supplementary file 5 [file Image_4.TIF]
